# Information gaps across care interfaces in home care and patient safety incidents: a cross-sectional survey of home care professionals in Finland

**DOI:** 10.1136/bmjopen-2026-121950

**Published:** 2026-10-26

**Authors:** Inka Sylgren, Esa Jämsen, Paulus Torkki

**Affiliations:** 1 Department of Public Health, University of Helsinki, Helsinki, Finland; 2 Department of Geriatrics, HUS Helsinki University Hospital, Helsinki, Uusimaa, Finland; 3 Faculty of Medicine, University of Helsinki, Helsinki, Finland; 4 Department of Industrial Engineering and Management, LUT University, Lappeenranta, Finland

**Keywords:** Multimorbidity, Aged, Hospital to Home Transition, Health Services for the Aged, Patient Care Management

## Abstract

**Abstract:**

**Objectives:**

To examine the occurrence and severity of information gaps across care interfaces in home care networks and to identify priority interfaces for improving information continuity and patient safety.

**Design:**

Cross-sectional survey study.

**Setting:**

Four publicly funded home care organisations in Finland, covering approximately 22% of Finland’s long-term home care clients nationally.

**Participants:**

555 home care professionals engaged in direct client care, including practical nurses, registered nurses, physicians and physiotherapists.

**Primary outcome measures:**

Frequency of information gaps across 11 predefined care interfaces (reported as always, often, sometimes, seldom or never) and severity of associated patient safety incidents (near miss, minor/moderate or severe) in the past 3 months, reported by the participants based on their experience.

**Results:**

The number of professionals working across the analysed interfaces ranged from 326 to 441. Information gaps were reported across all 11 interfaces, with those occurring always or often most frequently associated with emergency departments (21.5%), hospital discharge (19.1%) and private health centre (16.9%). Patient safety incidents were identified across all interfaces, particularly in relation to hospital discharge (44.1%) and emergency department (40.4%). Severe adverse events were most frequently reported in emergency care (4.3%), informal care (3.7%) and hospital discharge (3.4%), indicating variation in both occurrence and severity across interfaces.

**Conclusions:**

Information gaps and related patient safety incidents may concentrate at time-critical transitions but extend across the wider care network including informal care. These findings highlight system-level vulnerabilities and the need to prioritise safety-critical interfaces and strengthen information continuity in home care.

**STRENGTHS AND LIMITATIONS OF THIS STUDY**

- This study examined information gaps across 11 care interfaces simultaneously in organisations covering over one-fifth of Finnish home care clients, enabling a network-level perspective on information continuity rather than focusing on transitions.
- The use of healthcare professionals’ self-reported experiences enables identification of information gaps not visible in administrative or incident-reporting data, but may introduce recall and perception bias, and findings reflect professional perceptions rather than objectively verified incidents.
- The self-selection sampling approach, low response rate and heterogeneity of included interfaces may limit generalisability and obscure finer-grained differences between interfaces, while their potential influence on comparisons between interfaces remains uncertain.

## Background

 The growing number of older adults living with multimorbidity and cognitive decline has intensified the demand for care systems capable of addressing complex and multidimensional needs.^1–4^ Rather than being delivered by a single provider or organisation, such care typically unfolds across extensive networks of formal and informal health and social care providers.^5–7^ Spanning multiple professionals and organisational boundaries, these networks increase coordination demands,^6^ treatment burden^8^ and the risk of adverse events.^9 10^

Information continuity is fundamental for maintaining coordinated care within such complex networks.^11–13^ When relevant information is missing, delayed, inaccurate or inaccessible at the point of care, information gaps arise that disrupt care processes and may compromise patient safety.^14–16^ Research on information continuity in practice has largely focused on transitions and handovers,^17 18^ particularly emergency care^19–21^ and hospital discharge.^22–24^ While prior research has identified vulnerabilities in information continuity in specific settings and single care transitions, it has not examined how information gaps are distributed across the broader network of interfaces surrounding the care of multimorbid individuals, nor how their occurrence and severity shape the risk associated with these interfaces.

This limitation is particularly consequential in home care, where clients are highly vulnerable to even minor information breakdowns that can contribute to adverse events.^25–27^ In practice, this vulnerability is reflected in the everyday decision-making of home care professionals. Although care decisions are made across multiple services, home care professionals implement and manage them in patients’ everyday environments, often under conditions of incomplete or fragmented information.^28–30^ This may lead to three types of patient safety incidents: near misses, no-harm incidents and harmful incidents, the latter including adverse events and adverse reactions,^31^ which are presented in figure 1.

**Figure 1:**
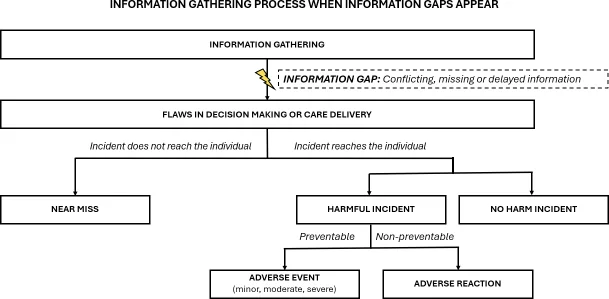
Conceptual framework linking information gaps to patient safety incidents. This framework illustrates how information gaps may lead to flawed decision-making and care delivery, which in turn can result in patient safety incidents, including near misses, no harm incidents and harmful incidents. Harmful incidents can be further divided into categories, including potentially preventable adverse events. The framework is modified from the WHO classification of patient safety incidents.^31^

To address patient safety incidents at a system level, this study adopts a network-based perspective encompassing interfaces between home care and other health and social care services.^5 32^ In this study, we use the term care interface to refer to a point of interaction between home care and other parts of the care network, including health and social care services and informal care, where care responsibilities and information exchange meet. By examining both the frequency of information gaps and the severity of associated patient safety incidents, interfaces that may warrant particular attention in improvement efforts can be preliminarily identified. Considering these two dimensions together may provide an initial indication of interfaces that could be particularly relevant for improvement efforts.

This study contributes to the literature by (1) extending the analysis of information continuity from single care transitions to a network of care interfaces, (2) providing an operational approach to characterise interface-level risk based on the occurrence of information gaps and the severity of associated patient safety incidents and (3) illustrating how this approach can be used to identify priority interfaces for improvement across home care networks. These contributions are addressed through the following research questions:

**RQ1** How frequently do information gaps occur between home care and other health and social care interfaces?

**RQ2** What is the severity of patient safety incidents associated with these information gaps?

**RQ3** Which interfaces may warrant particular attention in improvement efforts based on the frequency of information gaps and the occurrence of associated patient safety incidents?

## Methods

### Study setting

This was a cross-sectional survey study conducted in Finnish home care, which forms part of Finland’s universal welfare system. Home care is an integrated social and health service, organised by well-being service counties, and it is widely used among older adults; 13% of individuals aged ≥75 years and 29% of those aged ≥85 years used the service regularly in 2025.^33^ Among the regular clients, 59% received visits at least daily, and 19% received three visits per day.

The clients are characterised by high levels of dependency and clinical complexity. National data indicate that 77% of long-term clients experience cognitive decline, and 44% have diagnosed dementia.^34^ Care is delivered across multiple organisations and professionals, with medical care provided either by dedicated home care physicians or by general practitioners at local health centres. In addition to routine home care services, clients have both planned and unplanned contacts with other health and social care services, including emergency departments. For example, 30% of home care clients have experienced a fall within the past 3 months, and 13% have used emergency care services.^34^ Informal caregivers, family members and relatives are often involved in the care of clients with cognitive impairment.

### Data collection and participants

The study used a cross-sectional online survey conducted between May and September 2024. The sample was drawn from four publicly funded home care organisations covering approximately 22% of Finland’s long-term home care clients. Eligible participants included all professionals engaged in direct client care, such as practical nurses, registered nurses, physicians and physiotherapists. This inclusive approach was chosen to capture the multidimensional nature of information continuity across the entire professional network. The number of potential participants in each region ranged from 450 to 1700, with approximately 3650 eligible participants in total.

The study employed a self-selection sampling approach, in which all eligible professionals in the participating organisations were invited to participate through internal communication channels. The final study size was determined by the number of respondents who chose to participate; no formal sample size calculation was performed.

The survey was available online for 4 weeks. Response activity was monitored throughout the period, and reminders were circulated during weeks two and three to encourage participation. Respondents received no financial compensation but were permitted to complete the questionnaire during working hours. Data collection and management were conducted using REDCap software (V.14.5.25).

### Survey instrument

This study used a subset of items from a broader survey instrument on health and care information management in home care. The full instrument comprised 72 items, of which 32 were included in this study, including 10 items on demographic characteristics. The remaining items used ordinal response scales. Responses were required for all items; however, partially completed questionnaires were retained in the analysis.

The survey instrument was developed based on earlier qualitative findings.^15^ We refined the instrument through six cognitive interviews using a think-aloud approach, in which participants completed the survey while describing how they understood the questions and terminology. Following the think-aloud assessment, a short discussion was held about the instrument’s clarity, structure, comprehensibility and response options. The interviews included two professionals from each of the three main occupational groups (practical nurses, registered nurses and physicians). Based on the interviews, introductory text, terminology and question wording were clarified, examples of patient safety incidents were added to severity categories, and certain response options were modified. Moreover, the original five-category severity classification was revised to four categories to improve clarity and distinction between response options. Participants were also asked about the survey’s length, clarity and suitability for their professional role. The revised instrument was subsequently pilot-tested by two testers, and the average completion time was measured.

#### Information gap occurrence and adverse events

The main variables in this study included the occurrence of information gaps across care interfaces, the frequency of these gaps and the severity of associated patient safety incidents, as well as respondents’ demographic and work-related characteristics. We selected 11 different interfaces relevant to the client’s care based on our earlier study,^15^ relevant literature, and considering also clinical, practical and organisational aspects. The interfaces included home care physician, emergency department, public health centre (eg, general practitioner), private health centre (eg, private medical clinics providing general practitioner and specialist physician services), public specialised healthcare (eg, hospital outpatient clinics), hospital inpatient care, hospital discharge (client returning home), diagnostics, rehabilitation services and social services. In addition, information gaps between home care and relatives, family members or other informal caregivers (hereafter referred to as informal care) were included. Examples and brief descriptions were provided in the survey to support consistent interpretation of the predefined interfaces, particularly where categories could potentially overlap (see online supplemental A). Patient safety incidents were assessed using established patient safety severity classifications.

Respondents answered the same set of questions for each of the 11 interfaces. First, respondents were asked how often they had encountered an information gap between home care and the interface during the past 3 months. Response options were ‘Always (almost always; hereafter ‘Always’)’, ‘Often’, ‘Sometimes’, ‘Seldom’ and ‘Never’. Respondents who did not work with a given interface selected ‘Not applicable’. A detailed description of the survey instrument is provided in online supplemental A.

If the response was anything other than ‘Never’ or ‘Not applicable’, respondents were asked whether they had experienced any of the following: (i) near miss, (ii) minor or moderate patient safety incident or (iii) severe patient safety incident. The assessment was based on the respondent’s own experience and judgement of the consequences of the information gap, rather than on independent verification of the incident. Each was reported using the options ‘Yes’, ‘No’ or ‘I do not know’. After responding, participants proceeded to the next interface.

The severity categories were adapted from a patient safety incident reporting system (Haipro,^35^ which uses the same categorisation of patient safety incidents as the WHO’s (2020) classification.^31^ Definitions and short examples were provided for each category to guide respondents in assessing the consequences of the information gap (see online supplemental A). The ‘I do not know’ option was provided to allow responses also when the respondent was unable to determine. Potential sources of bias were addressed by limiting the recall period to 3 months, using standardised response options, refining the survey instrument through cognitive interviews and inviting all eligible professionals to participate.

#### Demographic characteristics

Respondents were asked to provide information on their demographic and work-related characteristics. Participants were asked to share their profession (practical nurse, registered nurse, therapist such as nutritionist or physiotherapist, physician or other including assisting personnel), age group (<30, 30–39, 40–49, 50–59, ≥60 years) and years of professional experience (<5, 5–9, 10–14, 15–19, 20–24, ≥25 years). They were also asked to report their gender (female, male, other or not disclosed).

Work-related characteristics included whether respondents engaged in client work, and if so, conducted in-person client work >3 days/week or online client work >3 days/week. Additionally, participants were asked about their employment status and whether they held a supervisory role.

### Data analysis

Descriptive statistical methods were used, including frequencies, percentages and 95% confident intervals (CIs) to summarise the occurrence of information gaps and associated patient safety incidents across care interfaces. The analysis proceeded in three stages. First, respondents working with each interface were identified, and analyses were conducted separately for all 11 interfaces, excluding responses marked as ‘Not applicable’. The original ordinal response categories (‘Always’, ‘Often’, ‘Sometimes’, ‘Seldom’ and ‘Never’) were retained in the descriptive analyses. For combined group analyses, ‘Always’ and ‘Often’ were combined to represent frequent information gaps, whereas ‘Sometimes’ and ‘Seldom’ were combined to represent occasional information gaps; ‘Never’ remained a separate category.

Second, we examined the proportion of respondents reporting at least one patient safety incident and the frequency of each incident type. Third, we attempted to prioritise the interfaces by combining information regarding occurrence and severity. We calculated the number of respondents who reported at least one patient safety category and the number who reported both minor or moderate and severe adverse events.

As responses were required for all items, item-level missing data were minimal; partially completed questionnaires were retained. Analyses were conducted using Microsoft Excel (V.2507; Build 19029.20208). No weighting was applied as there was no predefined or empirically established basis for assigning relative weights to frequency and severity.

### Patient and public involvement

Patients and members of the public were not involved in this study.

## Results

### Characteristics of study participants

In total, 555 respondents completed the survey (table 1). The number of respondents varied between 91 and 211 across the 4 regions, corresponding to a response rate of 12.4–20.2% with a weighted average of 15.2%. Practical nurses comprised 65.6% of participants, registered nurses 20.0% and other professional groups smaller shares. The largest age groups were 50–59 years (28.5%) and 40–49 years (24.3%). Work experience varied widely: 18.4% had <5 years and 18.7% had ≥25 years. Most respondents were female (90.1%), employed full-time (92.1%) and engaged in in-person client work (81.4%).

**Table 1:** Participant demographics and interface involvement

Variable	Total
	n (%)
Respondents	555 (100.0)
Region A	91 (16.4)
Region B	121 (21.8)
Region C	132 (23.8)
Region D	211 (38.0)
Profession	
Practical nurse	364 (65.6)
Registered nurse	111 (20.0)
Physician	16 (2.9)
Therapist (nutritionist, physiotherapist)	13 (2.3)
Other (assisting personnel, other)	51 (9.2)
Age	
<30	76 (13.7)
30–39	122 (22.0)
40–49	135 (24.3)
50–59	158 (28.5)
≥60	64 (11.5)
Years of experience	
<5	102 (18.4)
5–9	120 (21.6)
10–14	106 (19.1)
15–19	58 (10.5)
20–24	65 (11.7)
≥25	104 (18.7)
Gender	
Female	500 (90.1)
Male	43 (7.7)
NA (non-binary, prefer not to say)	12 (2.2)
Client facing work at home care	
Client work (yes)	514 (92.6)
In-person client work>3 days/week (yes)	452 (81.4)
Remote client work>3 days/week (yes)	62 (11.2)
Role-related status	
Full-time employment (yes)	511 (92.1)
Supervisor role (yes)	39 (7.0)
Respondents working with each interface	
Emergency department	441 (79.5)
Hospital discharge	440 (79.3)
Informal care	438 (78.9)
Public health centre	402 (72.4)
Rehabilitation	402 (72.4)
Home care physician	392 (70.6)
Hospital inpatient care	382 (68.8)
Specialised healthcare	381 (68.6)
Social services	370 (66.7)
Diagnostics	367 (66.1)
Private health centre	326 (58.7)

The number of respondents working with each care interface with home care varied from 326 to 441 of the 555 respondents (table 1). Most of the respondents worked in the interfaces of emergency department (79.5%, n=441), hospital discharge (79.3%, n=440) and with informal care (78.9%, n=438). While collaboration with private healthcare providers, diagnostic services and social services was least frequent, over half of the respondents were involved in these interfaces (58.7%, 66.1% and 66.7%, respectively).

### Distribution and occurrence of information gaps across interfaces

Information gaps were reported across all 11 care interfaces, although their occurrence varied substantially between interfaces (figure 2). Among respondents working with the respective interfaces, 30.8–73.6% reported information gaps during the past 3 months, with the highest proportion in relation to hospital discharge and the lowest in rehabilition.

**Figure 2:**
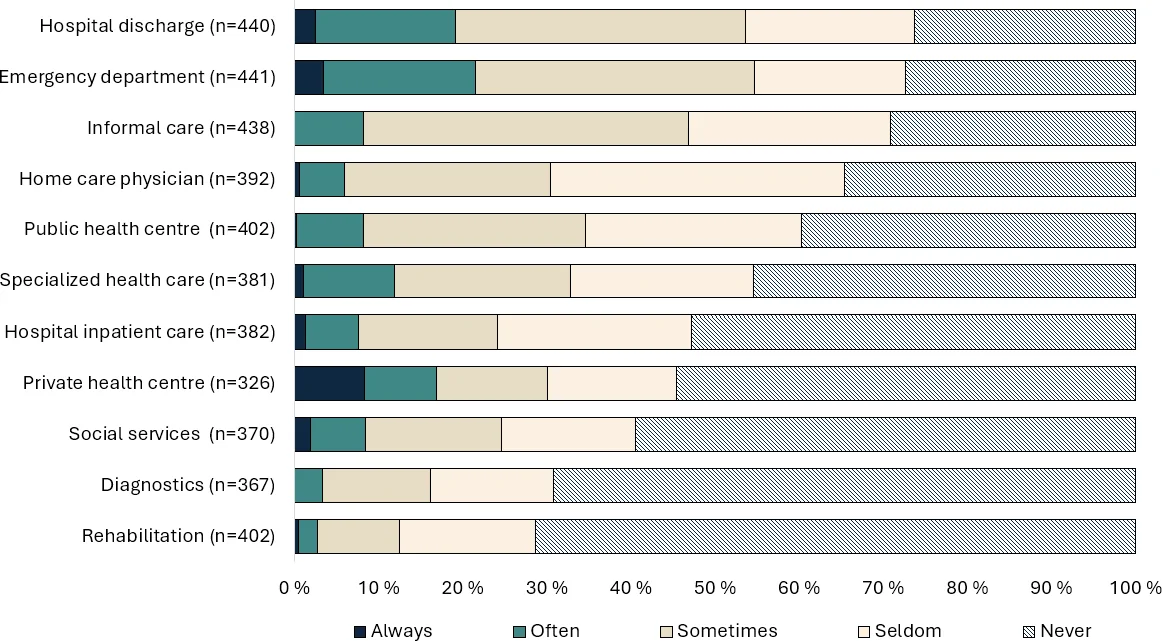
Occurrence of reported gaps during the past 3 months. Interfaces are ordered by the proportion of respondents reporting information gaps at least seldom (‘Always’, ‘Often’, ‘Sometimes’ or ‘Seldom’) in descending order. The number of respondents working in each interface is reported in parentheses.

Information gaps reported as occurring always were relatively uncommon. The highest proportion was reported for private health centres (8.3%, n=27), followed by emergency departments (3.4%, n=15) and hospital discharge (2.5%, n=11).

Frequent information gaps (always or often) were most reported in emergency departments (21.5%, n=95), hospital discharge (19.1%, n=84) and private health centres (16.9%, n=55). Occasional information gaps (sometimes or seldom) were common across several interfaces, particularly those involving informal care (62.6%, n=274), home care physicians (59.4%, n=233), hospital discharge (54.5%, n=240), public health centres (52.0%, n=209) and emergency departments (51.0%, n=225).

Information gaps were most commonly reported as never occurring in rehabilitation (71.4%, n=287), diagnostics (69.2%, n=254) and social services (59.5%, n=220). A detailed distribution of response categories across interfaces is provided in online supplemental B1 and B2.

### Severity of associated patient safety incidents

Participants reported patient safety incidents throughout the care network varying from 8.5% to 44.1% per interface in the past 3 months (table 2). Across interfaces, incident severity followed a similar hierarchical pattern: near misses were reported most frequently, followed by moderate and minor adverse events, while severe incidents were the least frequent. However, the relative proportions varied between interfaces.

**Table 2:** Reported patient safety incidents and their severity

		*Respondents reporting patient safety incidents*		*Patient safety incident types*		
**Interfaces**	**Respondents working with the interface, n**	**Respondents reporting ≥1 patient safety incident type,** **n (%**)	95% CI*	**Severe adverse events,** **n (%**)	**Minor or moderate adverse events,** **n (%**)	**Near misses,** **n (%**)
Hospital discharge	440	194 (44.1)	39.5 to 48.8	15 (3.4)	98 (22.3)	173 (39.3)
Emergency department	441	178 (40.4)	35.9 to 45.0	19 (4.3)	106 (24.0)	148 (33.6)
Public health centre	402	123 (30.6)	26.3 to 35.3	8 (2.0)	59 (14.7)	104 (25.9)
Home care physician	392	116 (29.6)	25.3 to 34.3	11 (2.8)	68 (17.3)	98 (25.0)
Informal care	438	125 (28.5)	24.5 to 32.9	16 (3.7)	123 (28.1)	NA
Specialised healthcare	381	101 (26.5)	22.3 to 31.2	4 (1.0)	53 (13.9)	90 (23.6)
Private health centre	326	70 (21.5)	17.4 to 26.2	4 (1.2)	35 (10.7)	62 (19.0)
Social services	370	62 (16.8)	13.3 to 20.9	2 (0.5)	31 (8.4)	57 (15.4)
Hospital inpatient care	382	62 (16.2)	12.9 to 20.3	4 (1.0)	29 (7.6)	55 (14.4)
Diagnostics	367	44 (12.0)	9.1 to 15.7	2 (0.5)	24 (6.5)	38 (10.4)
Rehabilitation	402	34 (8.5)	6.1 to 11.6	2 (0.5)	16 (4.0)	28 (7.0)

* CI calculated using the Wilson score method. All percentages represent the proportion of respondents working with each interface who reported the respective patient safety incident or severity category, using the number of respondents working with that interface as the denominator. Reported patient safety incidents represent respondents who reported at least one patient safety incident at the respective interface . NA due to a technical error in the survey instrument during data collection.

CI, Confidence interval; NA, not available.

The highest proportions of respondents reporting at least one patient safety incident type were at interfaces involving hospital discharge (44.1%, n=194), emergency care (40.4%, n=178), public health centres (30.6%, n=123), home care physicians (29.6%, n=116) and informal care (28.5%, n=125). In contrast, the lowest proportions of respondents reporting adverse events were reported at interfaces with rehabilitation (8.5%, n=34), diagnostics (12.0%, n=44) and hospital inpatient stays (16.2%, n=62).

The combined proportion of severe and minor or moderate adverse events was highest at interfaces involving informal care (n=139, 31.7%), emergency department (n=125, 28.3%), hospital discharge (n=113, 25.7%) and home care physicians (n=79, 20.2%), indicating both a high frequency of events and a greater burden of severe outcomes at these interfaces.

Across interfaces, the descriptive pattern showed that interfaces with higher proportions of near misses also tended to have higher proportions of minor or moderate and severe adverse events. The highest proportion of near misses was reported at hospital discharge (n=173, 39.3%), whereas the lowest was reported in rehabilitation services (n=28, 7.0%). Further details on the reported patient safety incidents and adverse events are provided in online supplemental table C1.

### Prioritisation of interfaces based on combined occurrence and severity

Figure 3 illustrates how the interfaces compare in terms of the occurrence of information gaps reported always or often and the proportion of respondents reporting at least one minor, moderate or severe adverse event. Emergency department and hospital discharge were located in the upper-right area of the figure, indicating both frequent information gaps and a higher proportion of adverse events. Informal care showed a relatively high proportion of adverse events despite a lower occurrence of information gaps. Rehabilitation, diagnostics and hospital inpatient care were positioned in the lower-left area, indicating lower levels of both indicators.

**Figure 3:**
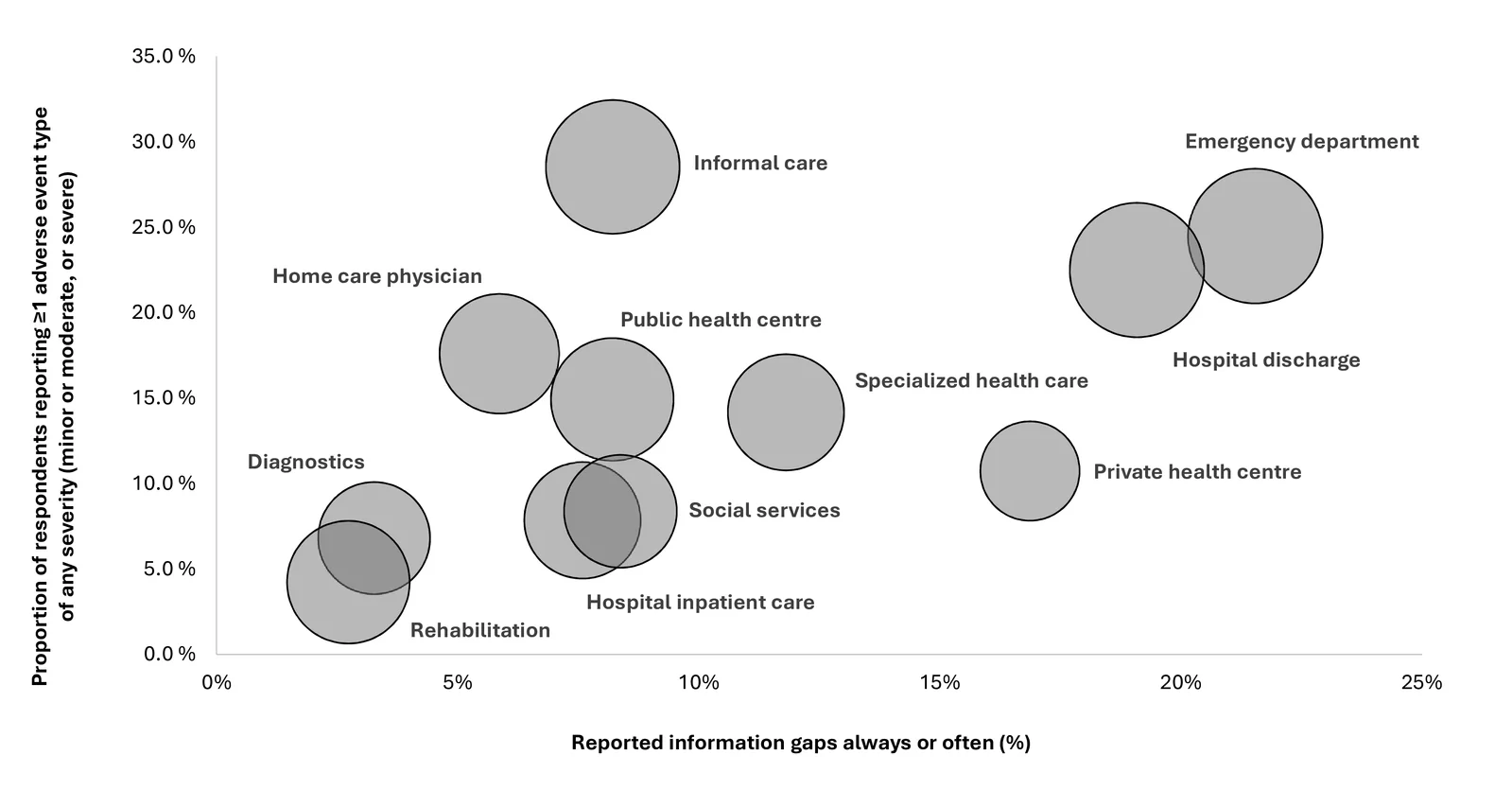
Information gaps and associated adverse events across care interfaces. Each point represents an interface. The horizontal axis shows the proportion of respondents working with each interface who reported information gaps ‘Always’ or ‘Often’, and the vertical axis shows the proportion who reported a severe or minor or moderate adverse event. For both proportions, the number of respondents working with each interface was used as the denominator and is represented by the bubble size. Interfaces with higher proportions on both axes were considered to warrant greater attention in improvement efforts.

## Discussion

### Information gaps in home care form a network-level patient safety risk across care interfaces

Examining the occurrence and severity of information gaps across care interfaces in home care networks, the findings suggest that information gaps and related patient safety incidents are not limited to single care transitions but represent a recurring phenomenon across multiple care interfaces surrounding home care. Although information gaps were reported across all examined interfaces, their occurrence and severity varied considerably, indicating that risks related to information gaps are unevenly distributed within the care network. By examining occurrence and severity across interfaces, this study introduces a network perspective that extends prior research beyond single interface transitions^14 20 24 36 37^ and patient safety incidents.^16 38–40^ This perspective is particularly relevant in multimorbid care, where information continuity links fragmented services and supports coordination across changing care actors.^5 13 41^

Taken together, these characteristics are reflected in differences in both the occurrence and severity of information gaps across interfaces, forming the basis for distinguishing four interface types with different risk profiles (figure 3). Three characteristics may potentially help explain these patterns: the level of support needed in care transfer, the extent of care alterations and the degree of time sensitivity. Interfaces such as emergency department visits and hospital discharge to home care may involve high support needs, rapid care alterations and strong time pressure. These situations may require continuous alignment between multiple actors and may rely on informal practices to compensate for fragmented information. Risks may increase when care plans must be adjusted quickly based on incomplete or delayed information. In contrast, more elective and less time-sensitive interfaces, such as rehabilitation or diagnostic testing, may typically involve fewer immediate changes to care and allow more time to interpret and integrate information, reducing both the likelihood and impact of information gaps.

#### High support needs in care transfer with urgent and direct care alteration: hospital discharge, emergency department

Hospital discharge and emergency department visits emerged as the interfaces where information gaps were most frequently reported, with potential implications for patient safety. Around one in five respondents reported frequent information gaps, and over one-third reported near misses, while minor and moderate adverse events were also frequent. These interfaces also showed the highest rates of severe adverse events, suggesting that they probably warrant particular attention in efforts to improve information continuity.

Previous research similarly identifies transitions from hospitals or emergency departments as vulnerable points in the healthcare system.^16 21 23 24 36^ Information generated during hospitalisation often fails to transfer effectively to post-acute care providers, leaving home care services to reconstruct care plans from incomplete discharge documentation and information obtained from patients and family members.^42 43^ At the same time, discharge and emergency department visits may involve rapid changes to medications, treatment plans and care needs, which may increase the need for support in care transfer between hospital and community providers.^14 20^ In such situations, incomplete or delayed information may influence subsequent care decisions.^19^ These situations may therefore be characterised by high support needs in care transfer, where both responsibility and information must be transferred rapidly and reliably.

From a system perspective, these findings highlight challenges in maintaining information continuity across organisational boundaries, where providers responsible for subsequent care may have limited access to earlier clinical information.^16 44^

#### High support needs in care transfer with indirect care alteration mediated informally: informal care

Interactions with informal caregivers were as frequent as those related to hospital discharge and emergency departments. However, while information gaps at this interface were reported less frequently, interactions involving informal caregivers were associated with comparatively higher proportions of adverse events. Importantly, near miss data were not available for the informal care interface due to a technical error in the data collection, which limits the comparability of this interface with the others and warrants caution when interpreting its patient safety profile.

There are several possible mechanisms that may contribute to these results. Informal caregivers play a central role in supporting older adults living at home by maintaining contextual knowledge and helping to bridge fragmented care systems through coordination and information flow across providers and settings.^5 13 32 45^ Moreover, family members may have an essential role in supporting daily care, for example, by administering medications or recognising changes in a home care client’s health status. Informal caregivers may have limited capacity to manage such tasks, which may increase the risk of miscommunication and adverse events.^46^

Despite their central role, informal caregivers are rarely integrated into formal care processes and typically have limited access to care documentation or shared electronic health records,^45 47^ and they are rarely supported with formal mechanisms for participating in information exchange. This may help explain why information gaps at this interface, although less frequent, were associated with comparatively severe consequences among the reported adverse events. Considering the important role of family caregivers,^48^ this interface warrants attention and further research is needed to establish the extent and nature of information gaps and patient safety risks associated with informal care.

#### Sustained support needs in care transfer with non-urgent but direct care alteration: private health centre, specialised healthcare, public health centre, home care physician

Interfaces with home care physicians, public health centres, specialised healthcare and private health centres involve medical decisions that modify ongoing care, typically outside urgent situations. While all involve physicians, their roles differ: home care physicians and public health centres often carry broader responsibility for overall care management, whereas specialised and private providers more frequently address specific diseases or treatments.

Communication between home care services and physicians has been described as relying on fragmented information exchange across organisational and technological boundaries.^5 22^ Studies of home care practice further show that nurses often need to assemble information from multiple sources or contact physicians to clarify treatment plans or medication changes, as documentation may be incomplete and information systems between providers are not always interoperable.^29 49^

Private care is used less frequently, but information gaps may arise when care is modified by providers not responsible for overall care and using separate health record systems. In such cases, changes to treatment or medication may not be fully communicated to home care services, requiring professionals to verify or reconstruct information across sources.

#### Lower support needs in care transfer with episodic and indirect care alteration: social services, hospital inpatient care, diagnostics and rehabilitation

Social services, elective hospital inpatient stays, diagnostics and rehabilitation represent interfaces where care interactions tend to be more episodic and where alterations to care delivered by home care are typically indirect. These interfaces may involve lower support needs in care transfer, as information exchange may be less time-critical and may not immediately alter care delivery. At these interfaces, providers may generate new information about the patient’s condition, functional ability or care needs, but this information does not necessarily lead to immediate modifications of everyday care routines. In our study, these were also the least frequently recognised interfaces in respondents’ daily work and were associated with the lowest occurrence of information gaps and patient safety incidents.

Previous research has highlighted that patient information exchange in community and home care should include a holistic understanding of the patient, covering not only medical conditions but also functional ability, psychosocial circumstances and everyday care needs. However, documentation systems often prioritise clinical information, while contextual information related to functional status or social situation may be less consistently documented.^30 49^ Information generated in services such as rehabilitation or social care may not always be fully visible in documentation despite its relevance for longer-term care planning. Given the low occurrence and severity reported, these interfaces may represent lower priorities for improvement.

### Strengths and weaknesses of the study

A key strength of this study is its system-level perspective on information gaps, achieved by examining multiple care interfaces simultaneously rather than focusing on single transitions and by including four regions that together account for over one-fifth of home care clients in Finland. The use of healthcare professionals’ self-reported experiences enables the identification of information gaps not visible in administrative or incident-reporting data, although the heterogeneity of included interfaces may obscure finer-grained differences. Importantly, the reported patient safety incidents reflect respondents’ reported experiences and perceptions and were not independently verified; therefore, the findings should not be interpreted as estimates of actual adverse event rates or as evidence of causal relationships between information gaps and patient harm.

Several factors may have influenced the reported occurrence of information gaps and related safety incidents. Less salient incidences and near misses may have been more difficult to recall, whereas more memorable experiences may have been more likely to be reported. Because participation was voluntary and response rates were relatively low, self-selection may have influenced the composition of respondents. Professionals with greater exposure to information gaps or a stronger interest in patient safety may have been more likely to participate, potentially leading to overestimation of their occurrence. Also, selection based on demographic or professional characteristics could not be assessed. Differences in the number and composition of respondents across interfaces may also have contributed to the observed variation between interfaces, although this effect may have been reduced by the fact that most respondents worked across multiple care interfaces.

Finally, the study reflects healthcare professionals’ perspectives and does not include patient or informal caregiver perspectives. Organisational differences between the four participating regions may also have influenced the reported patterns and limit generalisability. In addition, near miss data were unavailable for the informal care interface due to a technical error, limiting its comparability with other interfaces.

### Implications for practice

The findings point to an important implementation challenge. Although many high-risk transitions in the care pathway are already well recognised,^31^ preventable adverse events persist. Our findings indicate that reported information gaps and perceived patient safety incidents were particularly concentrated in emergency department visits and hospital discharge, suggesting that these interfaces may warrant prioritisation for improvement. In these interfaces, key challenges relate to ensuring that the right information reaches the right provider at the right time. Missing or incomplete information on medication, baseline status, care plans and responsibilities may lead to misinterpretation or omissions. However, the present study did not assess which specific information elements should be prioritised or standardised. Improving information continuity may therefore require both standardised communication and clearly defined coordination structures.^36 50 51^

The recurrence of similar gaps across interfaces suggests that these challenges are systemic and require a broader network perspective. Although the study was conducted in Finnish home care, the findings are likely relevant to other health and social care systems characterised by fragmented care pathways and multiple service providers. However, transferability may be more limited in countries where home care is organised through private, community-based or otherwise less integrated structures rather than through publicly organised home care. Greater structural fragmentation may increase the number of interfaces across which information must be transferred, potentially creating additional opportunities for information gaps. At the same time, differences in organisational responsibilities and information systems may result in different patterns of information gaps and patient safety risks.

### Future research

Future research should move beyond single transitions to examine multiple care interfaces across care journeys, including the role of informal care. Incorporating perspectives from patients and informal caregivers could provide complementary insights into information continuity across these interfaces. Our findings suggest that persistent information gaps may reflect limitations in how interfaces are specified in practice, particularly regarding what information is transferred, in what format and with what level of responsibility. Research should therefore prioritise developing and evaluating standardised information transfer practices in high-risk interfaces, such as emergency departments and hospital discharge and examine how coordination structures shape information exchange. Also, combining survey-based evidence with incident-reporting data or care record reviews could further strengthen understanding of information gaps and their potential patient safety implications.

## Conclusion

The findings suggest that information continuity challenges in home care may be patterned rather than sporadic, with information gaps reported particularly at interfaces characterised by high coordination demand, direct care alteration and time sensitivity. Interfaces where care direction changes rapidly or where responsibility is distributed across multiple actors may be particularly prone to information gaps associated with more serious patient safety consequences. By adopting a network-based perspective on care pathways, the findings offer a basis for identifying potentially high-risk interfaces and considering targeted, interface-specific improvements to reduce patient safety incidents.

## Supplementary material

10.1136/bmjopen-2026-121950 online supplemental file 1

## Data Availability

Data are available upon reasonable request.
