# Supplementary material for: Information gaps across care interfaces in home care and patient safety incidents: a cross-sectional survey of home care professionals in Finland
Source: BMJ Open. 2026 Oct 26;16(10):e121950. doi: 10.1136/bmjopen-2026-121950 (PMC13711515; doi:10.1136/bmjopen-2026-121950)
Supplement: Supplementary file 1 [file bmjopen-16-10-s001.pdf]

## SUPPLEMENTARY MATERIALS

### A1 Structure and logic of the survey instrument

For each interface, respondents first reported the occurrence of information gaps. If they had experienced a discontinuity, they were then asked whether it resulted in a near miss, a mild or moderate adverse event, or a severe adverse event.

### Care interfaces, their order and additional notes for clarification

1. Home care physician
2. Emergency department
3. Public health centre (e.g., public general practitioner)
4. Private health centre (e.g., private general practitioner)
5. Specialised health care (e.g., outpatient clinic visit)
6. Hospital at discharge (client returning home)
7. Hospital inpatient care
8. Laboratory, radiology, or other diagnostic services
9. Rehabilitation services
10. Social services (e.g., housing, mental health, substance use services)
11. Informal care (a person who plays a significant role in care)

**Step 1.**

**How often have you encountered information gaps between home care and** [interface] **during the past three months?**
*Note. If you do not work with the interfaces, choose "Not applicable"*

Options: Always (almost always) / Often / Sometimes / Seldom / Never / Not applicable

If the response was Always, Often, Sometimes, or Rarely, respondents proceeded to 2a-c.

If the response is **Never / Not applicable**, respondents proceeded to the next interface.

**Steps 2a-c.**

**Did the information gap result in any of the following?**
Options for each item: Yes / No / I do not know

- **A near miss**
  A situation in which a potentially harmful event was identified and prevented before harm occurred, but required additional work to clarify the issue or prevent harm (e.g., clarification of diagnostic orders or medication changes made elsewhere).
- **A minor or moderate adverse event**
  Harm resulting from care delivered based on incorrect or incomplete information, leading to prolonged care, additional interventions, or patient suffering.
- **A severe adverse event**
  Serious harm such as hospitalisation, permanent injury, life-threatening event, or death.

**Example of the Survey Instrument**

| Step | Component | Question | Response Options | Conditional logic |
|---|---|---|---|---|
| **1** | Occurrence (screening question) | How often have you encountered an information gap between home care and [care interface] during the past three months? | Always (almost always)<br><br>Often<br><br>Sometimes<br><br>Seldom<br><br>Never<br><br>Not applicable | If **Never** or **Not applicable →** survey proceeds to next interface.<br><br>If answer is **Always / Often / Sometimes / Seldom** → Steps 2a-c shown |
| **2a** | Near miss | Did the information gap result in a near miss? | Yes<br><br>No<br><br>I do not know | |
| **2b** | Minor or moderate adverse event | Did the information gap result in a mild or moderate adverse event? | Yes<br><br>No<br><br>I do not know | |
| **2c** | Severe adverse event | Did the information gap result in a severe adverse event? | Yes<br><br>No<br><br>I do not know | |

**…**

**B1. Distribution of answers of each interface**

| Interface | Always (n, % of n) | Often (n, % of n) | Sometimes (n, % of n) | Seldom (n, % of n) | Never (n, % of n) | Total (n, % of n) |
|---|---|---|---|---|---|---|
| Diagnostics | 0 (0.0) | 12 (3.3) | 47 (12.8) | 54 (14.7) | 254 (69.2) | 367 (100.0) |
| Emergency department | 15 (3.4) | 80 (18.1) | 146 (33.1) | 79 (17.9) | 121 (27.5) | 441 (100.0) |
| Home care physician | 2 (0.5) | 21 (5.4) | 96 (24.5) | 137 (34.9) | 136 (34.7) | 392 (100.0) |
| Hospital discharge | 11 (2.5) | 73 (16.6) | 152 (34.5) | 88 (20.0) | 116 (26.4) | 440 (100.0) |
| Hospital inpatient care | 5 (1.3) | 24 (6.3) | 63 (16.5) | 88 (23.0) | 202 (52.9) | 382 (100.0) |
| Informal care | 0 (0.0) | 36 (8.2) | 169 (38.6) | 105 (24.0) | 128 (29.2) | 438 (100.0) |
| Private health centre | 27 (8.3) | 28 (8.6) | 43 (13.2) | 50 (15.3) | 178 (54.6) | 326 (100.0) |
| Public health centre | 1 (0.2) | 32 (8.0) | 106 (26.4) | 103 (25.6) | 160 (39.8) | 402 (100.0) |
| Rehabilitation | 2 (0.5) | 9 (2.2) | 39 (9.7) | 65 (16.2) | 287 (71.4) | 402 (100.0) |
| Social services | 7 (1.9) | 24 (6.5) | 60 (16.2) | 59 (15.9) | 220 (59.5) | 370 (100.0) |
| Specialized health care | 4 (1.0) | 41 (10.8) | 80 (21.0) | 83 (21.8) | 173 (45.4) | 381 (100.0) |

**B2. Distribution of answers of each interface with combined categories**

| Interface | Always or often, (n, % of n) | Sometimes or seldom, (n, % of n) | Never, (n, % of n) | Total (n, % of n) |
|---|---|---|---|---|
| Diagnostics | 12 (3.3) | 101 (27.5) | 254 (69.2) | 367 (100.0) |
| Emergency department | 95 (21.5) | 225 (51.0) | 121 (27.5) | 441 (100.0) |
| Home care physician | 23 (5.9) | 233 (59.4) | 136 (34.7) | 392 (100.0) |
| Hospital discharge | 84 (19.1) | 240 (54.5) | 116 (26.4) | 440 (100.0) |
| Hospital inpatient care | 29 (7.6) | 151 (39.5) | 202 (52.9) | 382 (100.0) |
| Informal care | 36 (8.2) | 274 (62.6) | 128 (29.2) | 438 (100.0) |
| Private health centre | 55 (16.9) | 93 (28.5) | 178 (54.6) | 326 (100.0) |
| Public health centre | 33 (8.2) | 209 (52.0) | 160 (39.8) | 402 (100.0) |
| Rehabilitation | 11 (2.7) | 104 (25.9) | 287 (71.4) | 402 (100.0) |
| Social services | 31 (8.4) | 119 (32.1) | 220 (59.5) | 370 (100.0) |
| Specialized health care | 45 (11.8) | 163 (42.8) | 173 (45.4) | 381 (100.0) |

### C1. Respondents reporting patient safety incidents and adverse events across interfaces

| Interface | Respondents working with the interface, n | Respondents reporting ≥1 minor/moderate or severe adverse event, n (%) | Respondents reporting ≥1 patient safety incident type, n (%) |
|---|---|---|---|
| Diagnostics | 367 | 25 (6.8) | 44 (12.0) |
| Emergency department | 441 | 108 (24.5) | 178 (40.4) |
| Home care physician | 392 | 69 (17.6) | 116 (29.6) |
| Hospital discharge | 440 | 99 (22.5) | 194 (44.1) |
| Hospital inpatient care | 382 | 30 (7.9) | 62 (16.2) |
| Informal care | 438 | 125 (28.5) | 125 (28.5) |
| Private health centre | 326 | 35 (10.7) | 70 (21.5) |
| Public health centre | 402 | 60 (14.9) | 123 (30.6) |
| Rehabilitation | 402 | 17 (4.2) | 34 (8.5) |
| Social services | 370 | 31 (8.4) | 62 (16.8) |
| Specialized health care | 381 | 54 (14.2) | 101 (26.5) |
