# Supplementary material for: Information gaps across care interfaces in home care and patient safety incidents: a cross-sectional survey of home care professionals in Finland
Source: BMJ Open. 2026 Oct 26;16(10):e121950. doi: 10.1136/bmjopen-2026-121950 (PMC13711515; doi:10.1136/bmjopen-2026-121950)
Supplement: Supplementary file 2 [file bmjopen-2026-121950.reviewer_comments.pdf]

PEER REVIEW HISTORY

BMJ Open publishes all reviews undertaken for accepted manuscripts. Reviewers are asked to complete a checklist review form and are provided with free text boxes to elaborate on their assessment. These free text comments are reproduced below.

ARTICLE DETAILS

Title (Provisional)

Information gaps across care interfaces in home care and associated adverse events: a cross-sectional survey of home care professionals in Finland

Authors

Sylgren, Inka; Jämsen, Esa; Torkki, Paulus

VERSION 1 - REVIEW

**Reviewer**	**1**
**Name**	**Kalra, Saurabh**
**Affiliation**	**University of Miami Miller School of Medicine, Public Health Sciences**
**Date**	**24-May-2026**
**COI**	**None**

This is an important and timely manuscript addressing information continuity and patient safety in home care networks. The network-based perspective across multiple care interfaces is novel and clinically relevant, particularly given the increasing complexity of multimorbid older adults receiving fragmented care. Please find comments below that can improve the flow and transparancy.

1. The authors acknowledge self-selection sampling, but the discussion of its implications remains limited. It would strengthen the manuscript to discuss:

* Whether respondents differed systematically from the underlying workforce,

* Whether professionals experiencing more information gaps may have been more likely to participate,

* How this may influence prevalence estimates and interface prioritization.

2. The study relies entirely on descriptive statistics. While exploratory analyses are acceptable, the manuscript repeatedly compares interfaces and implies meaningful differences in risk profiles without formal statistical testing. Consider adding confidence intervals, comparative analyusese, sensitivity analyses, etc At the minimum limitations.

3. The concept of "interface-level risk" is interesting and potentially novel, but remains somewhat conceptual rather than rigorously operationalized. The prioritization framework in Figure 3 is visually useful, but the rationale for combining frequency and severity is not fully

justified methodologically. The manuscript would benefit from clear explanatiuon, whether any weighting was considered, why serious and moderate events were combined.

4. Some interfaces may overlap conceptually or operationally (e.g., public health centres, home care physicians, specialized health care). It is unclear how respondents distinguished among these categories in practice. Additional clarification examples needed

5. Some parts of the Discussion infer mechanisms that were not directly measured (e.g., "rapid care alterations," "time pressure," "support needs in care transfer"). These are plausible interpretations but should be presented more cautiously as hypotheses rather than established explanations.

6. Consider discussing generalizability outside Finland in more detail, particularly to healthcare systems without integrated public home care structures.

**Reviewer**	**2**
**Name**	**Muwanguzi, Patience A.**
**Affiliation**	**Makerere University**
**Date**	**30-Jul-2026**
**COI**	**None**

Thank you for the opportunity to review this manuscript. I found the topic important and very relevant to patient safety, home care and the care of older people with multimorbidity. I particularly appreciated the decision to examine information gaps across 11 care interfaces rather than focusing only on hospital discharge or emergency department transitions. This gives the paper a useful network-level perspective and makes the findings potentially valuable for service planning and quality improvement.

Title and abstract

1. The title is clear and accurately identifies the study design. The abstract is well structured, but the results need to be checked carefully against the tables. Some percentages in the abstract do not appear to match the main results.
2. Please check the figures for patient safety incidents and severe adverse events. For example, the abstract reports patient safety incidents at hospital discharge as 59.9%, but Table 2 reports 44.1%. The abstract also reports severe adverse events at hospital discharge as 5.2%, while Table 2 reports 3.4%. These differences may be due to different denominators, but this is not clear to the reader.
3. I would also encourage the authors to state clearly in the abstract that the findings are based on professionals' self-reported experiences. This is important because the study does not verify incidents through clinical records or incident-reporting systems.

Background

4. The background is strong and provides a clear rationale for the study.

The authors explain why information continuity matters in complex home care networks and why home care clients may be vulnerable to harm from fragmented information. I especially appreciated the move from individual transitions to a broader network-based perspective.

5. My suggestion here is to define “interface” earlier and more simply. The definition appears later, but the paper would benefit from a clearer explanation at the start, since this is one of the central concepts of the manuscript.
6. The final paragraph of the background is helpful because it states the study contribution and research questions clearly. This is well done.

Methods

7. The cross-sectional survey design is appropriate for describing the frequency and perceived severity of information gaps across interfaces. The description of the Finnish home care setting is also helpful.
8. The survey instrument needs more detailed explanation. The authors state that the instrument was based on earlier qualitative findings and tested through six cognitive interviews. Please explain what the cognitive interviews assessed, what changes were made after testing, and whether the final instrument was assessed for clarity, content validity or acceptability.
9. Please also clarify how the 11 interfaces were selected. Were these entirely based on the previous qualitative study, expert input, policy relevance, or common home care workflows? This matters because the whole analysis depends on these predefined interfaces.
10. The response rate is low, ranging from 12.4% to 20.2%, with a weighted average of 15.2%. I appreciate that survey response rates can be difficult in busy care settings, but this raises a concern about self-selection bias. Professionals who had experienced information gaps or patient safety concerns may have been more likely to respond. Please discuss this more clearly in the methods or limitations.
11. The section on patient safety incidents also needs clarification. Respondents were asked whether an information gap resulted in a near miss, minor/moderate adverse event, or severe adverse event. Please explain how respondents were expected to make this judgement. Were definitions and examples enough to support consistent interpretation? Were respondents reporting confirmed incidents, suspected incidents, or perceived consequences?

Data analysis

12. The descriptive analysis is broadly appropriate. However, the authors should consider adding confidence intervals to the main proportions, especially where interfaces are compared. This would help readers understand the uncertainty around the estimates.
13. Please also explain why “always” and “often” were grouped together. This is sensible, but the rationale should be stated. Similarly, please explain why minor/moderate and severe adverse events were combined in the prioritisation analysis.
14. My concern with Figure 3 is that “per employee” is not clearly explained. Please clarify whether the denominator is all respondents,

respondents working with each interface, or another denominator. The figure is potentially very useful, but it needs a clearer explanation in the text and caption.

Results

15. The results address the research questions and the findings are important. However, the current presentation has several inconsistencies that need careful correction. First, the heading on page 9 appears incomplete: "Characteristics of study participants and". Please revise.
16. Please harmonise all denominators across Table 1, Figure 2 and Supplement B. For example, Table 1 reports 402 respondents working with rehabilitation, while Figure 2 reports rehabilitation as n=317. Table 1 reports informal care as n=438, while Supplement B reports n=530. Similar inconsistencies appear for specialised health care, public health centre and social services. This is my biggest concern in the results because the reader cannot easily know which denominator is correct.
17. Please revise the wording around severe adverse events on page 12. The sentence says that the highest proportions of respondents reporting at least one severe adverse event were hospital discharge at 44.1% and emergency care at 40.4%. However, Table 2 shows that these percentages refer to any reported patient safety incident, not severe adverse events. Severe adverse events are reported separately as 3.4% and 4.3%.
18. Please be careful when combining categories. Table 2 states that respondents could select one or more severity categories. If so, adding severe and minor/moderate categories together may double count respondents unless the authors calculated unique respondents who selected either category. Please clarify this.
19. Figure 3 needs a more detailed caption explaining the axes, the bubble size, the denominator and how "priority" should be interpreted. Table 2 should use "patient safety incidents" rather than "patient safety incidences."
20. For Figure 1, please ensure that terminology aligns with the text and WHO classification. The figure uses "no harmful incident," while the text uses "no-harm incident." Please standardise.

Discussion

21. The discussion is thoughtful and shows good engagement with the literature. I appreciated the attempt to classify interfaces according to support needs, care alteration and time sensitivity. This is one of the more interesting contributions of the paper.
22. However, my concern here is that some interpretation goes beyond what the study design can support. This was a self-reported cross-sectional survey with a low response rate. The study can show that respondents reported information gaps and perceived patient safety incidents across interfaces. It cannot confirm actual adverse event rates or causal relationships between information gaps and patient harm.

23. Please soften statements such as “This study demonstrates” and “information continuity challenges are systematically patterned.” Suggested wording would be “The findings suggest” or “Respondents’ reports indicate.”
24. The discussion of informal care is important and well placed. However, because near-miss data for informal care were not available due to a technical error, this should be handled more cautiously. The paper should not overinterpret the informal care interface without fully acknowledging this missing data problem.

Strengths and limitations

25. The strengths are well stated. The limitations need more detail. Please expand this section to include the low response rate, self-selection bias, recall bias, perception bias, lack of objective verification of incidents, absence of patient and informal caregiver perspectives, possible organisational differences across the four regions, and the technical error affecting near-miss data for informal care.
26. The statement that the limitations are “unlikely to substantially affect comparisons” feels too confident. I would soften this because the denominator inconsistencies, low response rate and self-selection bias may affect comparisons between interfaces.

Implications and future research

27. I agree that emergency department visits and hospital discharge appear to be priority areas for improvement. The manuscript would be stronger if the authors made the recommendations more specific. For example, what information should be standardised at discharge? Medication changes, baseline function, care plan changes, escalation instructions, follow-up responsibilities and informal caregiver roles could be named.
28. The future research section is appropriate. I would add that future work should include patient and informal caregiver perspectives and, where possible, link survey findings to incident-reporting data or care record reviews.

VERSION 1 - AUTHOR RESPONSE

R#	*Reviewer comment*	*Author response*	*Line(s)*
R1.0	This is an important and timely manuscript addressing information continuity and patient safety in home care networks. The network-based perspective across multiple care interfaces is novel and clinically relevant, particularly given the increasing complexity of multimorbid older adults receiving fragmented care. Please find comments below that can improve the flow and transparancy.	-	-

R1.1	The authors acknowledge self-selection sampling, but the discussion of its implications remains limited. It would strengthen the manuscript to discuss:<br>- Whether respondents differed systematically from the underlying workforce,<br>- Whether professionals experiencing more information gaps may have been more likely to participate,<br>- How this may influence prevalence estimates and interface prioritization.	We have expanded the Strengths and weaknesses section and discussed more broadly the potential self-selection bias, including its possible effects on reported occurrence and interface prioritisation. We also note that, as respondents commonly assessed multiple interfaces, self-selection is unlikely to fully explain the observed differences between interfaces. See also R2.10, R2.26.	436-446
R1.2	The study relies entirely on descriptive statistics. While exploratory analyses are acceptable, the manuscript repeatedly compares interfaces and implies meaningful differences in risk profiles without formal statistical testing. Consider adding confidence intervals, comparative analyusese, sensitivity analyses, etc At the minimum limitations.	We have added 95% confidence intervals to the main proportions in Table 2, particularly for the interface-specific estimates, to better illustrate the uncertainty around the estimates. See also R2.12. softened the wording used to interpret the results in the Discussion section. See comments R2.22-R2.26	213-215, 223-226
R1.3	The concept of "interface-level risk" is interesting and potentially novel, but remains somewhat conceptual rather than rigorously operationalized. The prioritization framework in Figure 3 is visually useful, but the rationale for combining frequency and severity is not fully justified methodologically. The manuscript would benefit from clear explanatiuon, whether any weighting was considered, why serious and moderate events were combined.	We agree that the framework is primarily conceptual and descriptive. We have therefore revised the terminology and clarified the rationale for the prioritization analysis. Rather than combining severity categories, the revised analysis compares frequent information gaps with the proportion of respondents reporting at least one patient safety incident. We did not apply weighting to frequency or severity. As there was no predefined or empirically established basis for assigning relative weights to these dimensions, introducing weights would have required assumptions about their relative importance and could have resulted in an arbitrary prioritization of care interfaces. We also acknowledge that the framework may provide a basis for further methodological development and validation.	90-93, 223-224, 229-230
R1.4	Some interfaces may overlap conceptually or operationally (e.g., public health centres, home care physicians, specialized health care). It is unclear how respondents distinguished among these categories in practice. Additional clarification examples needed	The interfaces were selected to reflect the organisation of health services in Finland and were considered readily recognisable to healthcare professionals. Although hospital discharge and hospital inpatient care partly overlap, they represent distinct phases of the patient care process. Rehabilitation is conceptually broader, as it may take place in various types of units; however, the descriptions provided in the survey supported its consistent interpretation in the intended	175-177

		context. The distinctions between the interfaces were further clarified during the cognitive interviews, and participants did not report difficulties in differentiating between them.	
R1.5	Some parts of the Discussion infer mechanisms that were not directly measured (e.g., "rapid care alterations," "time pressure," "support needs in care transfer"). These are plausible interpretations but should be presented more cautiously as hypotheses rather than established explanations.	We have revised the discussion to present the proposed mechanisms, including care transfer needs, care alterations, and time sensitivity, as possible explanations rather than established mechanisms, using more cautious wording throughout.	326, 369
R1.6	Consider discussing generalizability outside Finland in more detail, particularly to healthcare systems without integrated public home care structures.	We have expanded the discussion of generalisability beyond Finland, particularly regarding healthcare systems where home care is less integrated with broader health and social care structures and where greater structural fragmentation may create additional interfaces for information exchange.	468-474
R2.0	Thank you for the opportunity to review this manuscript. I found the topic important and very relevant to patient safety, home care and the care of older people with multimorbidity. I particularly appreciated the decision to examine information gaps across 11 care interfaces rather than focusing only on hospital discharge or emergency department transitions. This gives the paper a useful network-level perspective and makes the findings potentially valuable for service planning and quality improvement.	-	-
R2.1	The title is clear and accurately identifies the study design. The abstract is well structured, but the results need to be checked carefully against the tables. Some percentages in the abstract do not appear to match the main results.	We carefully revised and harmonised the denominators across Table 2, Figure 2, and Supplement B1 and made the corresponding corrections throughout the manuscript. The inconsistencies in the reported numbers of respondents working with rehabilitation, informal care, specialised health care, public health centres, and social services have been corrected. The denominators are now consistent across all tables, figures, and supplementary materials.	Table 2, Figure 2, Supplement B1.
R2.2	Please check the figures for patient safety incidents and severe adverse events. For example, the abstract reports patient safety incidents at hospital discharge as 59.9%, but Table 2 reports 44.1%. The abstract also reports severe adverse events at hospital discharge as 5.2%,	We have revised the figures in the abstract to align with Table 2 and clarified in the Table 2 footnote that all percentages use the number of respondents working with each interface as the denominator.	35-39, Table 2, 276-281, 294-298

	while Table 2 reports 3.4%. These differences may be due to different denominators, but this is not clear to the reader.		
R2.3	I would also encourage the authors to state clearly in the abstract that the findings are based on professionals' self-reported experiences. This is important because the study does not verify incidents through clinical records or incident-reporting systems.	We have clarified in the abstract under the "Participants" heading that the findings are based on professionals' self-reported experiences.	31
R2.4	The background is strong and provides a clear rationale for the study. The authors explain why information continuity matters in complex home care networks and why home care clients may be vulnerable to harm from fragmented information. I especially appreciated the move from individual transitions to a broader network-based perspective.	-	-
R2.5	My suggestion here is to define "interface" earlier and more simply. The definition appears later, but the paper would benefit from a clearer explanation at the start, since this is one of the central concepts of the manuscript.	We agree that the term "interface" requires a clear definition at the beginning of the manuscript, and we have added a concise definition to the Background section.	88-90
R2.6	The final paragraph of the background is helpful because it states the study contribution and research questions clearly. This is well done.	-	-
R2.7	The cross-sectional survey design is appropriate for describing the frequency and perceived severity of information gaps across interfaces. The description of the Finnish home care setting is also helpful.	-	-
R2.8	The survey instrument needs more detailed explanation. The authors state that the instrument was based on earlier qualitative findings and tested through six cognitive interviews. Please explain what the cognitive interviews assessed, what changes were made after testing, and whether the final instrument was assessed for clarity, content validity or acceptability.	We have added additional information on the development and what type of changes were made during this process. See also E.2	150-162
R2.9	Please also clarify how the 11 interfaces were selected. Were these entirely based on the previous qualitative study, expert input, policy relevance, or common home care workflows? This matters because the whole analysis depends on these predefined interfaces.	We have clarified that the 11 interfaces were selected by the authors, primarily informed by findings from our previous qualitative study [15], while also drawing on relevant literature and the authors' clinical and health service system expertise. Practical considerations were also taken into account, as the number of	154-158

		interfaces needed to remain manageable. We therefore focused on interfaces considered to be among the most commonly encountered in home care while aiming to minimise overlap between them. Their relevance and clarity were subsequently discussed during cognitive interviews with professionals from different occupational groups. We also acknowledge that some potentially relevant interfaces, such as emergency medical services, may therefore have remained outside the scope of the study. See also Supplement A.	
R2.10	The response rate is low, ranging from 12.4% to 20.2%, with a weighted average of 15.2%. I appreciate that survey response rates can be difficult in busy care settings, but this raises a concern about self-selection bias. Professionals who had experienced information gaps or patient safety concerns may have been more likely to respond. Please discuss this more clearly in the methods or limitations.	We have expanded the discussion of potential self-selection bias. We have addressed this in the Strengths and weaknesses section. See also R1.1	463-446
R2.11	The section on patient safety incidents also needs clarification. Respondents were asked whether an information gap resulted in a near miss, minor/moderate adverse event, or severe adverse event. Please explain how respondents were expected to make this judgement. Were definitions and examples enough to support consistent interpretation? Were respondents reporting confirmed incidents, suspected incidents, or perceived consequences?	We have clarified that respondents assessed patient safety incidents based on their own experiences and judgement of the perceived consequences of information gaps. We also specified that definitions and examples were provided for each severity category to support consistent interpretation, and that the incidents were not independently verified through clinical records or incident-reporting systems.	187-189, 193-195
R2.12	The descriptive analysis is broadly appropriate. However, the authors should consider adding confidence intervals to the main proportions, especially where interfaces are compared. This would help readers understand the uncertainty around the estimates.	Thank you for this important comment. We have added 95% confidence intervals to the main proportions in Table 2 to better reflect the uncertainty around the estimates. Given the exploratory and descriptive nature of the study, we have retained the descriptive analytical approach and clarified this limitation in the manuscript. See also R1.2	213-215, Table 2
R2.13	Please also explain why “always” and “often” were grouped together. This is sensible, but the rationale should be stated. Similarly, please explain why minor/moderate and severe adverse events were combined in the prioritisation analysis.	Thank you for this comment. We have further clarified the grouping of response categories in the Methods and Results sections and added Supplement B2 to provide a more detailed presentation of the grouped response categories.	217-221, 223-226

R2.14	My concern with Figure 3 is that "per employee" is not clearly explained. Please clarify whether the denominator is all respondents, respondents working with each interface, or another denominator. The figure is potentially very useful, but it needs a clearer explanation in the text and caption.	We have clarified the denominator in both the text and the Figure 3 caption. For both proportions, the denominator is the number of respondents working with each respective interface. We have also removed the ambiguous term "per employee" and revised the caption accordingly. See also R2.19	Figure 3, 688-694
R2.15	The results address the research questions and the findings are important. However, the current presentation has several inconsistencies that need careful correction. First, the heading on page 9 appears incomplete: "Characteristics of study participants and". Please revise.	The word "and" has been removed from the heading. Other inconsistencies have also been addressed in accordance with the reviewers' comments.	235
R2.16	Please harmonise all denominators across Table 1, Figure 2 and Supplement B. For example, Table 1 reports 402 respondents working with rehabilitation, while Figure 2 reports rehabilitation as n=317. Table 1 reports informal care as n=438, while Supplement B reports n=530. Similar inconsistencies appear for specialised health care, public health centre and social services. This is my biggest concern in the results because the reader cannot easily know which denominator is correct.	We carefully revised and harmonised the denominators across Table 1, Figure 2, and Supplement B1 and made the corresponding corrections throughout the manuscript. The inconsistencies in the reported numbers of respondents working with rehabilitation, informal care, specialised health care, public health centres, and social services have been corrected. The denominators are now consistent across all tables, figures, and supplementary materials.	Table 1, Figure 2, Supplement B1.
R2.17	Please revise the wording around severe adverse events on page 12. The sentence says that the highest proportions of respondents reporting at least one severe adverse event were hospital discharge at 44.1% and emergency care at 40.4%. However, Table 2 shows that these percentages refer to any reported patient safety incident, not severe adverse events. Severe adverse events are reported separately as 3.4% and 4.3%.	Thank you for highlighting this inconsistency. We have corrected the percentages to match the correct ones.	276-281
R2.18	Please be careful when combining categories. Table 2 states that respondents could select one or more severity categories. If so, adding severe and minor/moderate categories together may double count respondents unless the authors calculated unique respondents who selected either category. Please clarify this.	We calculated the combined minor/moderate and severe adverse event category using unique respondents, so that respondents selecting both categories were counted only once. We have clarified this in the manuscript and added Supplement C1 to present the corresponding numbers of unique respondents.	Supplement C1, Table 2
R2.19	Figure 3 needs a more detailed caption explaining the axes, the bubble size, the denominator and how "priority" should be interpreted. Table 2 should use "patient safety incidents" rather than "patient safety incidences."	We have expanded the Figure 3 caption to clarify the axes, bubble size, denominator, and how priority should be interpreted. We have also corrected the terminology in Table 2. See also R2.14	Figure 3, Table 2

R2.20	For Figure 1, please ensure that terminology aligns with the text and WHO classification. The figure uses "no harmful incident," while the text uses "no-harm incident." Please standardise.	The terminology in Figure 1 and the accompanying text has been changed to "no harm incident" to align with the WHO classification.	84, Figure 1, 680-681
R2.21	The discussion is thoughtful and shows good engagement with the literature. I appreciated the attempt to classify interfaces according to support needs, care alteration and time sensitivity. This is one of the more interesting contributions of the paper.	-	-
R2.22	However, my concern here is that some interpretation goes beyond what the study design can support. This was a self-reported cross-sectional survey with a low response rate. The study can show that respondents reported information gaps and perceived patient safety incidents across interfaces. It cannot confirm actual adverse event rates or causal relationships between information gaps and patient harm.	Thank you for this important comment. We have revised the Discussion to more clearly acknowledge these limitations, including their implications for practice. We have also softened relevant statements throughout the Discussion and used more cautious wording when interpreting the findings.	286-287, 324-334, 432-435, 456-458
R2.23	Please soften statements such as "This study demonstrates" and "information continuity challenges are systematically patterned." Suggested wording would be "The findings suggest" or "Respondents' reports indicate."	We have also softened relevant statements throughout the Discussion and Conclusions sections and used more cautious wording when interpreting the findings. See comments R1.5 & R2.22	339-340, 343-344, 490-497
R2.24	The discussion of informal care is important and well placed. However, because near-miss data for informal care were not available due to a technical error, this should be handled more cautiously. The paper should not overinterpret the informal care interface without fully acknowledging this missing data problem.	We agree and have revised the Discussion to explicitly acknowledge the missing near miss data for informal care due to a technical error. We have also softened the interpretation of this interface and clarified that the observed adverse event pattern should be interpreted cautiously. Importantly, the technical error affected only the collection of near-miss data and did not affect the reporting of more severe adverse events for this interface.	365-368, 380-384
R2.25	The strengths are well stated. The limitations need more detail. Please expand this section to include the low response rate, self-selection bias, recall bias, perception bias, lack of objective verification of incidents, absence of patient and informal caregiver perspectives, possible organisational differences across the four regions, and the technical error affecting near-miss data for informal care.	We have expanded the Strengths and weaknesses section to provide more detail on the relatively low response rate and potential self-selection bias, recall and reporting bias, lack of independent verification of reported incidents, absence of patient and informal caregiver perspectives, organisational differences across the four regions, and the technical error affecting near-miss data for informal care. We have also clarified that the reported patient safety incidents reflect respondents' experiences and	426-451

		perceptions and that self-selection may have influenced the overall reported occurrence of information gaps and the observed differences between interfaces, although its impact on interface comparisons may have been limited because most respondents worked across multiple care interfaces.	
R2.26	The statement that the limitations are "unlikely to substantially affect comparisons" feels too confident. I would soften this because the denominator inconsistencies, low response rate and self-selection bias may affect comparisons between interfaces.	We agree that the previous wording was too direct. We have softened the statement in both the Strengths and weaknesses section and the Article summary to better acknowledge these limitations.	54-57, 436-446
R2.27	I agree that emergency department visits and hospital discharge appear to be priority areas for improvement. The manuscript would be stronger if the authors made the recommendations more specific. For example, what information should be standardised at discharge? Medication changes, baseline function, care plan changes, escalation instructions, follow-up responsibilities and informal caregiver roles could be named.	While we agree that such specific protocols might help prevent information gaps, this study did not examine the reasons for or mechanisms underlying the information gaps or patient safety incidents. The most appropriate solutions may also vary across regions and countries. For these reasons, the present findings do not provide a sufficient basis for specific recommendations, and the development and evaluation of such interventions would require further research.	461-462
R2.28	The future research section is appropriate. I would add that future work should include patient and informal caregiver perspectives and, where possible, link survey findings to incident-reporting data or care record reviews.	We have expanded the Future research section to acknowledge the value of patient and informal caregiver perspectives and the potential of combining survey findings with incident-reporting data or care record reviews.	478-480, 485-487

VERSION 2 - REVIEW

Reviewer 1

Name Kalra, Saurabh

Affiliation University of Miami Miller School of Medicine, Public Health Sciences

Date 19-Sep-2026

COI

Thank you for resolving my commments
